# Ethics, privacy and the legal framework governing medical data: opportunities or threats for biomedical and public health research?

**DOI:** 10.1186/0778-7367-71-15

**Published:** 2013-06-21

**Authors:** Yves Coppieters, Alain Levêque

**Affiliations:** 1 Research centre in epidemiology, biostatistics and clinical trials, School of Public Health, Université Libre de Bruxelles (ULB), Route de Lennik 808, CP 596, 1070, Brussels, Belgium

**Keywords:** Privacy, Medical data, Ethical considerations, Data analysis

## Abstract

Privacy is an important concern in any research programme that deals with personal medical data. In recent years, ethics and privacy have become key considerations when conducting any form of scientific research that involves personal data. These issues are now addressed in healthcare professional training programmes. Indeed, ethics, legal frameworks and privacy are often the subject of much confusion in discussions among healthcare professionals. They tend to group these different concepts under the same heading and delegate responsibility for “ethical” approval of their research programmes to ethics committees. Public health researchers therefore need to ask questions about how changes to legal frameworks and ethical codes governing privacy in the use of personal medical data are to be applied in practice. What types of data do these laws and codes cover? Who is involved? What restrictions and requirements apply to any research programme that involves medical data?

## Background

Privacy is an important concern in any research programme that deals with personal medical data. In recent years, ethics and privacy have become key considerations when conducting any form of scientific research that involves personal data [1]. These issues are now addressed in professional healthcare training programmes [2]. However, these programmes do not deal specifically with the legal framework or the procedures that apply to research involving medical data. Indeed, ethics, legal frameworks and privacy are often the subject of much confusion in discussions among healthcare professionals. They tend to group these different concepts under the same heading and delegate responsibility for the “ethical” approval of their research programmes to ethics committees.

This paper aims to give an overview of the main privacy and data protection issues that researchers need to take into account while working with health data.

## Results and discussion

In the European Union, there are no legal frameworks that aim to strike a balance between the individual right to privacy and the collective and social interest of research. The EU Data Protection Directive is the most important law when it comes to using personal (health) data [3]. The extent to which it is possible to use personal health data is determined by this law. This Directive is currently being revised, which opens up new possibilities but perhaps also new threats for public health monitoring and research.

Directive 95/46/EC3 was adopted in 1995 with two objectives in mind: to protect the fundamental right to data protection and to guarantee the free flow of personal data between Member States. It was complemented by Framework Decision 2008/977/JHA as a general instrument at Union level for the protection of personal data in the areas of police co-operation and judicial co-operation in criminal matters [3].

According to the definition of “data processing for statistical purposes”, however, this covers “any operation involving the collection and processing of personal data necessary for statistical research or the production of statistical results”. Article 4.5 of Recommendation R (97) 5 on the protection of medical data states that “medical data concerning unborn children should be considered as personal data and enjoy a protection comparable to the protection of the medical data of a minor” [4]. This document also contains recommendations covering the processing of genetic data [5].

As well as this European regulation, there are also national legal frameworks that aim to strike a balance between the individual right to privacy and the collective and social interest of research [6].

These laws often only cover a specific aspect of privacy – i.e. personal data – and only apply to certain types of individual, certain types of data processing, etc. Public health researchers therefore need to ask questions about how changes to legal frameworks and ethical codes governing privacy in the use of personal medical data are to be applied in practice. What types of data do these laws and codes cover? Who is involved? What restrictions and requirements apply to any research programme that involves medical data?

### Types of data and research concerned

Data concerning deceased individuals are in principle not protected and their use is not governed by legal frameworks. The Working Party on the protection of individuals with regard to the processing of personal data set up by Directive 95/46/EC of the European Parliament and of the Council of 24 October 1995 issued in this regard its interpretation of data of deceased persons: “Information relating to dead individuals is therefore in principle not to be considered as personal data subject to the rules of the Directive, as the dead are no longer natural persons in civil law. However, the data of the deceased may still indirectly receive some protection in certain cases” [7,8].

The majority of national legal frameworks only cover a specific aspect of privacy – i.e. personal data concerning individuals which are processed in some way. In order to be classed as “personal”, the data must not only refer to an individual but it must also be possible to identify the individual from such personal data (the one definition used within the EU legal data protection framework). In this respect, it is important to make a distinction between directly identifiable data and indirectly identifiable data. The implications for researchers are indeed not the same. If the data are indirectly identifiable through the codification of an independent organism, the constraints for the search should be decreased. However, this distinction is purely theoretical, as no such distinction is made in law between these two types of data. This distinction generally applies to electronic files rather than paper-based records.

Personal medical data contain medical information collected in the context of healthcare. Together, these data form an individual’s medical record. In most cases, this record is stored in electronic format for ease of transfer and dissemination. This raises the potential, however, for violations of privacy, including the risk that medical data may be used for purposes other than healthcare. The definition of “personal medical data” goes beyond information related solely to healthcare, however. It also covers personal data which, due to their nature or the way in which they are used, reveal information about an individual’s past, current or future physical or mental health [9].

In terms of data processing – i.e. any operation performed on data, such as collection, recording, organization, storage, adaptation or modification, extraction, consultation, use, transmission, dissemination or any other form of disclosure – it is important to make a clear distinction between personal data, key-coded data and anonymous data.

The use of anonymous data or key-coded data should not primarily be seen as a means to avoid risk of prosecution. The main concern is the protection of the privacy of an individual. If the researchers fail, then there is indeed a risk of prosecution. Moreover, medical information is sensitive information and by definition prohibited from being processed. Although the legislation might seem stringent and to hamper research, the main goal should still be to install ethical reflection. In such cases, it is wise to seek the assistance of a privacy inspectorate – such as an ethics committee, a privacy protection body or an independent technical organization – to check that the research programme complies with applicable privacy legislation. The relevant body is often specified in national laws.

National data protection laws indeed try to strike such a balance taking into account both personal and societal interests.

### Protection of personal medical data

In terms of data security, this involves the obligation in several national laws, to appoint a security advisor. The security advisor should always have all the information necessary to perform its duties properly and timely. There are national guidelines for information security of personal data that define the objectives of safety limits for each institution - legal person, business or government - which keeps, uses, processes or communicates personal data and whose treatment requires prior authorization.

Information security is the set of management measures that ensure the confidentiality, integrity and availability of all forms of information [both electronic (digital) form and on paper are preserved], in order to ensure continuity of information and keeping the possible consequences of information security incidents within an acceptable predefined level. “Management measures” refers to all measures relating to policy, procedures, guidelines, methods and organizational structures. These measures can be of a managerial, technical or management level.

### Rights of the individual and obligations incumbent on the controller

Under privacy laws, the individual to whom the data is related has a certain number of rights. Similarly, the controller has a certain number of obligations. Any form of use that fails to comply with these rights and obligations may therefore be considered illegal. The majority of these rights and obligations concern the protection of the individual’s fundamental rights and freedoms, and in particular their right to privacy. The individual also has the right to request information from the controller and to access his or her personal data. This provision allows individuals to monitor and limit how their data are processed, check the accuracy of data and ensure that their data are used in proportion and for acceptable purposes. Furthermore, individuals have the right to object to the use of their data, the right to rectify data, the right not to be subject to automatic decisions that are individual in character, and the right to appeal.

For example, an individual can correct data, request additional information on their use, or simply remove data (through their health data). In practical terms, a scientific researcher may be exempted from the obligation to inform the individual where it would be impossible or where it would require a disproportionate effort by the researcher to do so [10]. The health data are collected on a voluntary basis directly from the participants concerned so no authorization for carrying out health information system, but a recommendation to respect privacy protection rules.

### Roles and obligations incumbent on ethical and legal framework inspectorates

At present, any researcher who wishes to analyse personal medical data must seek approval from the relevant legal inspectorate (ethics committee, expert committee or national privacy protection body) [9]. These bodies are responsible for passing judgement on privacy-related matters concerning data processing activities and for handling complaints from individuals who believe that their privacy has been infringed. However, there is no official framework governing the procedures requested by research sponsors or funding providers, despite the fact that they are often considered the “controllers” (even where they sub-contract the actual data processing work to a team of external researchers). In many cases, these institutions require research controllers to comply with national laws, to demonstrate that their request is ethically acceptable and to disclose whether the data requested are anonymous or personally identifiable.

The definition and interpretation of the role and responsibility of controller and processor differ over time and across EU Member States. “The concept of data controller and its interaction with the concept of data processor play a crucial role in the application of Directive 95/46/EC, since they determine who shall be responsible for compliance with data protection rules, how data subjects can exercise their rights, which is the applicable national law and how effective Data Protection Authorities can operate” (Opinion 1/2010 on the concepts of “controller” and “processor” by the Working Party on the protection of individuals with regard to the processing of personal data set up by Directive 95/46/EC of the European Parliament and of the Council).

Notwithstanding the practical and institutional restrictions that it raises, privacy compliance represents a major opportunity for all personal data research programmes in both the public and private sectors. Some research companies are now developing their own codes of practice and ethics for their specific fields [11]. This field-specific approach is essential in ensuring that the company meets its legal data management obligations and that its research is conducted under a wider ethical framework that considers the rights of all stakeholders and offers maximum protection for personal (and often sensitive) data. This also means that researchers should, wherever possible, aim to work with anonymous or key-coded data [12].

A field-specific approach regarding procedures for dealing with data protection is necessary. ISO 9001 norm defines the requirements for the organization of a system of quality management.

## Conclusions

However, the current legal and regulatory situation does not encompass such a field-specific approach [13]. The questions raised in this paper could be set against the current review and reform of the EU legislation on privacy protection. The national data protection acts are not still based on the EU legal framework. In the EU and at national level, there are not enough legal frameworks that try to strike a balance between data protection for the individual, societal interests and support for research.

## Abbreviations

EU: European Union; ISO: International Organization for Standardization.

## Competing interests

The authors declared that they have no competing interest.

## Authors’ contributions

YC drafted the manuscript. AL commented the draft versions. Both authors read and approved the final manuscript.
